# Effect of Concept Mapping Education on Critical Thinking Skills of Medical Students: A Quasi-experimental Study

**DOI:** 10.4314/ejhs.v31i2.24

**Published:** 2021-03

**Authors:** Aslami Maryam, Dehghani Mohammadreza, Shakurnia Abdolhussein, Ramezani Ghobad, Kojuri Javad

**Affiliations:** 1 Education Development Center, Quality Improvement Education Research Center, Shiraz university of Medical Sciences, Shiraz, Iran; 2 Department of Immunology, school of medicine, Ahvaz Jundishapur university of Medical Sciences, Ahvaz, Iran; 3 Center for Educational Research in Medical Sciences (CERMS), Department of Medical Education, School of Medicine, Iran University of Medical Sciences (IUMS), Tehran, Iran

**Keywords:** Concept Map, critical thinking skills, Medical education, medical students

## Abstract

**Background:**

Fostering critical thinking (CT) is one of the most important missions in medical education. Concept mapping is a method used to plan and create medical care through a diagrammatic representation of patient problems and medical interventions. Concept mapping as a general method can be used to improve CT skills in medical students. The aim of this study was to explore the effect of concept mapping on CT skills of medical students.

**Methods:**

This quasi-experimental study was conducted on 100 second-year medical students which take an anatomy course. Participants were randomly assigned into a control group (lecture-based) and an intervention group (concept mapping). CT levels of medical students were assessed using the California Critical Thinking Skills Test. Data were analyzed using independent sample t-test.

**Results:**

Before intervention, CT scores of the intervention and control groups were 6.68 ± 2.55 and 6.64 ±2.74, respectively, and after intervention, they were 11.64 ±2.29 and 10.04 ± 3.11, respectively. Comparison of mean score differences for both groups before and after intervention demonstrated that CT scores in the experimental group significantly increased after intervention (P=0.021).

**Conclusions:**

Medical students who were taught through concept mapping showed an increase in CT scores, compared with those in the control group. Medical students require effective CT skills in order to make sound knowledge-based assessment and treatment choices during patient care. Therefore, instructors and planners of medical education are expected to apply this educational strategy for developing CT skills in medical students.

## Introduction

Concept mapping (CM) is a technique developed by Joseph in the 1970s for visualizing the relationships among different concepts. CM is among the academic teaching strategies that have proven to be useful, in the development of active learning. A great number of studies have been conducted on the effectiveness of CM in fostering critical thinking skills (1,2).

Critical thinking (CT) skill can be defined as “the ability to apply higher cognitive skills (e.g., analysis, synthesis, self-reflection, and perspective taking) and or the ability to be open-minded and intellectually honest”. Critical thinking skill is the most important skill every physician needs, because the complex nature of providing healthcare requires physicians to d gather data, integrate and act upon constantly. This skill plays an essential role in the physician's clinical decision making, which is important in ensuring diagnostic accuracy, appropriate patient management and patient outcomes (3,4).

CT is very important in the medical field because it is t what physicians use to prioritize and make key decisions that can save lives. CT skills of physicians can really mean the difference between someone's life and death. Deficits in CT among physicians have significant implications for patients, including misdiagnosis, delays in diagnosis, treatment errors, lack of patient centered care or recognition of changes in clinical status (5,6). United Nations Educational, Scientific and Cultural Organization (UNESCO) believes that CT provides students with an up-to-date training system. This is why medical universities and their educators need to develop a medical curriculum that will foster CT skills among medical students (7).

Currently, universities in Iran are still relying on old traditional methods to teach medical students. During the last decade in Iran, medical educators w experienced challenges in providing students with appropriate curricula. They suggested that Iranian medical universities need to change their curriculum from focus on conventional teaching methods. Conventional teaching methods are subject-based, and teachers try to achieve learning objectives through large group lectures. In Lecture Based Learning (LBL), students are passively exposed to factual knowledge and do not learn or apply concepts. However, medical education is moving away from teacher-centered approaches and is incorporating more active learning methods. Active learning process can help foster students' critical thinking skills (8–10).

As researchers were experiencing difficulty with the active learning methods, they were looking for a learning method that will allow students to retain large amounts of information, integrate critical thinking skills, and solve complex clinical problems. CM has been recognized as an effective educational tool that has been used for over 25 years, and a growing body of literature indicates that its t usage in medical education is increasing (11).

CM is a useful learning tool that creates the opportunity to promote CT skills by providing students opportunities to learn in a meaningful way. This method has helped students to score better on problem solving tests that require recall, transfer and application of knowledge. The map allows students to demonstrate holistic knowledge of a certain topic by showing how concepts on the map are interrelated (12,13).

CM is one of the most effective teaching strategies whose effect on promoting CT has frequently been explored and confirmed. It is a schematic system representing a set of concepts embedded in a framework of propositions. In other words, it is a diagram that shows multiple relationships among concepts (14). There are two prominent methods that medical students can use to create concept maps to promote meaningful learning. The first method requires students to construct their own maps by creating linking phrases between concepts. On the other hand, the other method, which is referred to as the scaffolding expert maps requires students to fill in blank spaces. This latter is effective for introducing students to CM. As it accurately reflects the knowledge structure of learners, it is more effective in demonstrating students' misunderstanding and misconceptions, it allows students to show how much they have learned, and it uses higher order cognitive processes, such as explaining and reasoning. Theorists believe the lower cognitive load associated with scaffold maps allows the learner to have a sharper focus on concepts involved (15).

Researchers have reported conflicting results about the effect of CM on increasing students' CT skills (16–18). Further studies are i required to clarify this issue. The purpose of this study was to determine if CM is more effective in teaching medical students anatomy topics compared to traditional lecturing method. We predict that students taught through concept map method will score better on California CT Test compared to students taught through traditional lecturing methods.

## Materials and Methods

**Study design and participants**: This was a two-group quasi-experimental study with a pre- and post-test design. Participants consisted of a total of 108 second-year medical students who were enrolled in Ahvaz Jundishapur University of Medical Sciences (AJUMS) attending an anatomy course in 2017. This course was offered in the third semester of the second-year program in a 12-week course on medical anatomy. None of the participants had previous experience in the use of CM in their curriculum. A research assistant explained the nature and purpose of the study for the participants. All medical students voluntarily took part and signed an informed consent. Codes were provided to participants for the demographic survey to ensure data confidentiality. Participants were provided with detailed verbal and written explanations of the study and were told that they could withdraw from the study at any time. They were also assured that their participation in the research would not affect their success in the course.

Before starting the course classes, the study sample (108 students) was randomly divided into two equal groups, 54 each. One group was considered as the intervention group (concept mapping) and the other was considered as the control group (lecture). The intervention group was taught through concept mapping, while the control group was taught by traditional didactic lecturing alone. The study was conducted for over 12 weeks, starting on September 2017. In the 1st week of the semester, pre-testing of the students' critical thinking skills in both experimental and control groups was done using California critical thinking Skills Test, form B (CCTST form B), before implementation of CM to identify their critical thinking level. It consisted of 34 multiple choice questions designed for the assessment of CT skills. In this test, each correct answer represents one score. The minimum and maximum obtainable scores are 0 and 34, respectively (19). In Iran, the reliability and validity of this test have already been determined and confirmed (20).

During the first week of the course, in a 2-hour session, the students in the intervention group were taught how to construct a concept map and how to use it appropriately in the context of anatomy. The students in the intervention group were required to present their discussion findings using the CM technique. Every student was required to prepare a concept map for all the presented topics. In each training session, the maps were assessed by the researcher, and the students were given feedback. Participants compared similarities and differences between their concept maps to assist in development of their individual concept map during their anatomy class. Training of the control group was done through traditional lecture method using power point software. The students of both groups passed 12 sessions of “anatomy course” in 12 consecutive weeks in lecture and CM methods, respectively.

**Figure 1:**
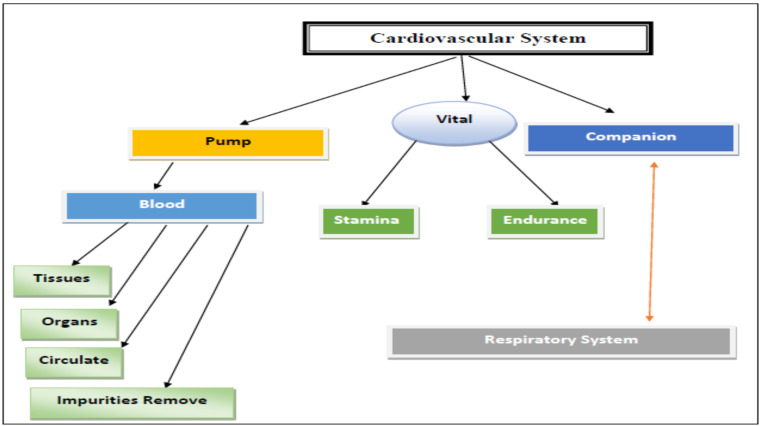
Student concept map. An example of a concept map from one of the medical students in this study

A concept map is a diagram that visually illustrates relationships between concepts and ideas. Concept maps are free of color and pictures, and are constructed in a top-to-bottom hierarchy. Most concept maps illustrate ideas as boxes or circles, which are structured hierarchically and connected with lines. These lines are labeled with linking words to help explain the connections between concepts. An example of a mind map created by a medical student in this study can be seen in Figure 1.

At the end of the semester in the 12th week, a post-test was conducted for both experimental and control groups by administering the CCTST (form B). The results of the pre- and the post-tests of the two groups were compared to assess the effect of using CM on increasing the students' critical thinking skills. To this aim, the difference between the mean of CT scores before and after intervention in each group was calculated and then the differences between the two groups were analyzed.

The room and time of the anatomy class were different for the two groups, but despite this, the students were in contact with each other in college and dormitory. Therefore, they may have exchanged concept map information with each other.

Data analysis was done using SPSS version 16. Descriptive statistics for some data such as gender, age and GPA was computed using frequencies, percentages, mean and standard deviation. Student characteristics were described, chi-square tests of differences between groups were conducted for categorical variables, and t-tests were performed for continuous variables. Two-group independent t-tests compared the CT scores at the beginning and end of the course between the two measurements.

## Results

Out of the 108 subjects participating in the study, 100 completed the questionnaire and returned it (response rate 92.6%). Table 1 presents the students' demographic characteristics. Subjects were homogenous in terms of gender, age and GPA. In the experimental group, 60% of subjects were females and 40% were males. In the control group, 54% were females and 46% were males. Sex distribution was similar in both groups (p=0.34). The mean age of medical students in both groups was also similar. In the experimental group, the mean age of subjects was 20.96 years (SD =0.88) and in the control group, it was 20.90 years (SD = 0.84). No significant differences were found between the control and experimental groups regarding age (p= 0.53). The grade point average (GPA) of previous semesters of experimental group was 15.20 (SD = 1.24) and that of the control group was 15.46 (SD = 1.17). In relation to students' GPA, chi-square test did not reveal a significant difference between the two groups (p=0.280).

**Table 1:** Students' characteristics in two different experimental and control groups

Variable	Control group	Experimental group	t	p. value
male	20(40%)	30(60%)	0.367	0.34
female	23(46%)	27(54%)		
Students' age	0.84 ±20.90	0.88 ±20.96	0.349	0.73
GPA of previous semesters	15.45± 1.17	1.24 ±15.20	1.09	0.28

**Figure 2:**
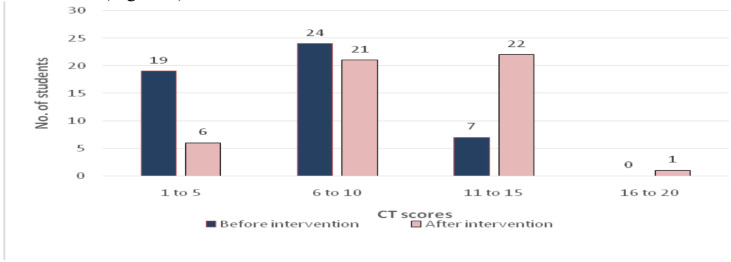
Critical thinking scores of the control group before and after intervention

The analysis scores of CT skills before intervention among both groups revealed that the mean CT scores of experimental and control groups were 6.64 and 6.68, respectively. The scores of experimental group before intervention ranged from 2 to 13 and that of the control group from 2 to 12 (Figure 2).

**Figure 3:**
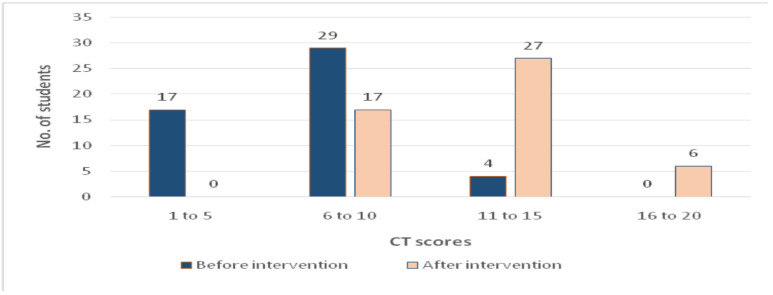
Critical thinking scores of the experimental group before and after intervention

The mean CT scores after intervention among the experimental and control group were found to be 10.04 and 11.64, respectively. The post intervention scores ranged from 8 to 17 in the experimental group and from 3 to 16 in the control group (Figure 3).

Table 2 demonstrates the students' CT skills by group. As shown in Table 2, before intervention, scores of CT skills in the intervention and control groups did not differ, which indicates that the students' performance was similar when they started the course. However, after intervention, CT scores were significantly higher in the intervention group. Students in the intervention group performed much better on the CT levels than students in the control group (P=0.004).

**Table 2:** Comparison of mean and SD of critical thinking skills in experimental and control groups before and after the intervention

Variable	Control group		Experimental group		t	p. value

	Mean	SD	Mean	SD		
Before intervention	6.64	2.74	6.68	2.55	0.076	0.94
After intervention	10.04	3.11	11.64	2.29	2.93	0.004
Difference	3.40	3.34	5.04	4.88	2.34	0.021

The average score obtained in anatomy course at the final exam by the entire medical student group was 5.98. The average score obtained in anatomy course by the experimental group was 6.38 and that of the control group was 5.58 (out of 10). There was a statistically significant difference between the two groups (t=2.67, p=0.009).

## Discussion

This study examined the impact of concept mapping on CT skills in the medical students in an anatomy course. The findings indicated that the use of CM had a positive effect on CT skills. This improvement could be achieved as a result of the teaching anatomy course using CM as a learning method. Researchers believe map construction is a useful tool that helps students to develop clinical reasoning skills through additional focus on logic, and it probably stimulates the use of thinking skills, such as analysis, interpretation, and evaluation, and finally promotes the development of CT skills (21).

The findings of our study are consistent with those of Kaddoura and Yang (2016) who analyzed the impact of a concept map teaching approach on nursing students' CT in the context of pathophysiology and pharmacology. Kaddoura and Yang integrated concept map teaching strategies in courses in order to develop CT skills in their students. They found that using CM in the education of nursing students leads to development of CT skills (22). A recent study was done by Sarhangi et al. on Iranian nursing students, indicating that the CM had a positive effect on CT skills (23). The result of a review article also indicated that CM affects the CT affective dispositions and CT cognitive skills (24).

Correspondingly, the findings of this study are also consistent with those of Nirmala et al. (2011), Deshatty et al. (2013), Moattari et al. (2014), Orique and McCarthy (2015), Mohamed (2017), and Elasrag (2020), who explored the effects of CM in promoting CT skills (2,14,25–28). These researchers concluded that CM is an effective strategy for improving students' ability to think critically.

In a systematic review on the use of CM in Iran, it was reported that CM had an important effect on improvement of critical thinking skills (29). The reason CT skills are promoted in CM is due to the fact that in this method, the learner has an active role in his/her own learning, which leads to promotion of high level learning. In this regard, these active interactions between learner/instructor and apparent organizing of the concepts allow the learner and instructor to exchange their perspective on how to communicate internal concepts, and they also would be able to discover missing concepts and communications, determine new educational needs, and restart the realignment of the map, which is the very self-assessment process that is part of the main CT skills (1). CM can be used in medical education in order to provide comprehensive and patient-centered care, prepare the medical students for clinical processes and make a connection between theory and clinical courses. Therefore, considering the advantages of this method in terms of increasing higher learning level and increasing medical students' motivation for learning, using this method in a more practical way in medical students' education is recommended.

However, our study findings are inconsistent with those of Bixler et al. (2015) who attempted to improve CT skill among fourth-year medical students using small group concept mapping (30). They found no significant increase in CT skills from pre-test to post-test when medical students were educated using a CM method. They proposed that the short time and the limited number of topics to which CM was applied may not provide a sufficient dose to impart a significant improvement on the CT Skills.

Our findings were also inconsistent with those of D'Antoniin et.al (2010) who conducted a quasi-experimental study to determine the effect of CM on CT skills in first-year medical students (31). They found no significant differences between the pre-test and post-test scores of the medical students for CT skills. D'Antoniin et al proposed that there may be a dose effect for using concept mapping, that is, more practice across more different kinds of scenarios may be required to equip students in this complex skill. Of course, this hypothesis requires further studies. Abdoli demonstrated that overall mean CT was not statistically significant after intervention for nursing students in their fourth semester at Isfahan University (32). He argued that perhaps one semester of using CM may not be sufficient to measure the effects of CM on the CT skill of students.

Researchers believe that absence of significance increase in overall CT scores after intervention might be derived from several factors related to the circumstances under which the study is conducted, the participants' conception and comfort level with participating in the study, seriousness in taking tests, the brevity of the experiment, and participants' developmental stages. In addition, it may be because that a training course alone is not significantly correlated with the critical thinking, because acquiring critical thinking skills needs a long period of time and continuing education (14,33).

Although there was a significant improvement in the CT skills among the experimental group, the overall scores of the experimental and the control groups were found to be very poor. The reason for the poor CT scores in students needs to be explored in a separate study. These findings were supported by Mohamed et al. (2017), who conducted a study for improving critical thinking of nursing students by the implementation of CM in Cairo University, Egypt. They reported low scores of critical thinking and added that low scores of critical thinking among their study subjects can be attributed to the educational system followed in secondary schools in Egypt (14). It is mainly a pedagogical approach with traditional teacher-centered rather than student-centered learning, where the student is mostly a passive recipient. Such a traditional educational approach does not foster CT skills in students.

The current study also revealed that CM not only leads to improved CT skills in medical students, but also causes a significant increase in students' scores in anatomy course. Indeed, anatomy topics can be effectively incorporated into concept map, making it easier for medical students to learn anatomy topics. Students being taught anatomy topics through concept map method had higher scores on anatomy course compared to students in the traditional lecturing group. This suggests that concept map is an effective educational tool and should be incorporated into medical education curriculum because it encourages students to become more independent learners and enhances learning of medical courses.

Improving high levels of thinking skills as one of the important missions of medical education makes it necessary to use appropriate approaches for developing CT skills. Most of the research conducted on the effectiveness of concept maps in medical education has focused primarily on the nursing population (24). However, our study included a sample of medical students. There are few studies similar to ours that have taught the intervention group concept map and then reevaluated students' CT skills. This study builds on previous work to suggest that CM is an effective strategy for developing medical students' ability to think critically. Using CM in the education of medical students appears to predict the improvement of CT skills, which is one of the most important missions of medical education. It is recommended that program directors and medical faculties evaluate their curricula to integrate concept map teaching strategies in other clinical courses to improve CT abilities in their students.

A limitation of this study was the small sample size and examining only one group of students (medical students). Another limitation was the use of only one course of medical program. In addition, since CM education is not used in other medical schools in Iran, the results of the study cannot be generalized to all medical students. We recommend that future researchers design a study with a larger sample size that includes other fields of medical sciences such as dentistry and pharmacy. Due to inability to keep interactions between the students during practicum under control, the students were likely to influence each other while creating concept maps. For future studies, it would be valuable to use a blinded study design to control for the interaction between experimental and control groups. This will help to reduce information exchange between both groups.

The finding of the current study showed an increase in CT scores in the experimental group as compared to the control group. Therefore, it can be concluded that CM strategy can promote critical thinking skills compared with traditional methods. Accordingly, instructors and curriculum planners are expected to apply this educational strategy for developing CT skills in medical students. The results also suggest that medical curricula need to change based on a student-centered learning approach.

The following recommendations are suggested as implications for future research:

- A longitudinal study is recommended that uses CM throughout the whole medical program curriculum, not just one course; it would be a more effective measure of the actual effect of CM on CT skill of medical students over time.
- In addition, it is recommended that a comprehensive study be replicated with a larger sample size, and randomly select from multiple different medical schools, in order to provide a more accurate representation of medical students.
- A number of participants withdrew during the second stage of the study due to the challenges with administering California Critical Thinking Questionnaire. Future research, should address the challenges associated with administering the California Critical Thinking Questionnaire in order to reduce participant attrition rate.
- Finally, we recommend researchers to explain the benefits and advantages of this study to participants prior to the start of their study.

This study was approved by Ethics Committee of Shiraz University of Medical Sciences (Ethics Code of: IR.SUMS.REC.F1202).
